# National Epidemiologic Survey of Thyroid cancer (NEST) in Korea

**DOI:** 10.4178/epih.e2018052

**Published:** 2018-10-26

**Authors:** Chang-Mo Oh, Hyun-Joo Kong, Eunyang Kim, Hyejin Kim, Kyu-Won Jung, Sohee Park, Young-Joo Won

**Affiliations:** 1 Cancer Registration and Statistic Branch, National Cancer Control Institute, National Cancer Center, Goyang, Korea; 2 Department of Preventive Medicine, Kyung Hee University School of Medicine, Seoul, Korea; 3 Department of Biostatistics, Graduate School of Public Health, Yonsei University, Seoul, Korea

**Keywords:** Thyroid neoplasms, Incidence, Mortality, Epidemiology, Korea

## Abstract

The Korea Central Cancer Registry conducted the National Epidemiologic Survey of Thyroid cancer (NEST) to investigate changes in the epidemiological and clinical characteristics of thyroid cancer patients between 1999 and 2008. The NEST was designed to collect representative samples of patients with thyroid cancer diagnosed in the years 1999, 2005, and 2008 using a proportionally stratified and systematic random sampling method. Among 42,891 participants diagnosed with thyroid cancer, 5,796 participants were included in the final study population. This survey collected information on diagnostic methods and date, route of diagnosis, prior medical history and history of thyroid-related disease, tumor, lymph node, metastasis and collaborative stage, and treatment. The NEST dataset was also linked to the cause-of-death database from Statistics Korea. The mean age of the study participants was 46.9 years. The ratio of men to women was 1:5.5. In the analysis of the histologic type of cancer, the proportion of papillary thyroid carcinoma showed an increasing trend (p<0.01). In contrast, the proportion of distant metastasis and the mean tumor size of thyroid cancers showed decreasing trends over time (p<0.01, respectively).

## INTRODUCTION

**Figure 1.**
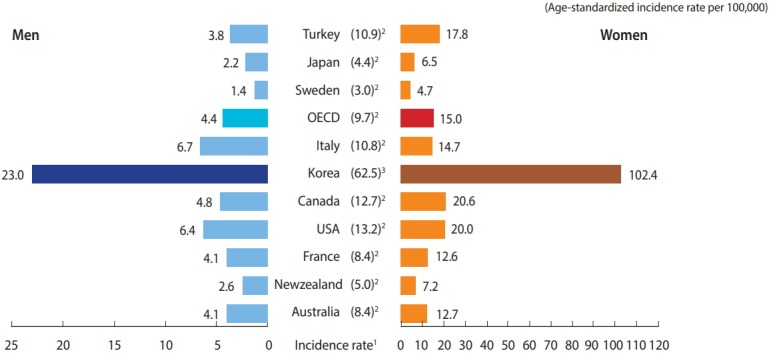
International comparisons of incidence rates of thyroid cancer. OECD, Organization for Economic Cooperation and Development. Adapted from: [1] The standard population per 100,000 people was calculated using Segi’s world standard population; [2] The estimated incidence rate in 2012 based on the data from GLOBOCAN 2012, International Agency for Research on Cancer, 2013; and [3] The observed incidence rate in year 2012 was obtained from the Korea Central Cancer Registry.

From 2009 to 2014, thyroid cancer was the most common cancer in South Korea (hereafter Korea). Korea had the highest incidence of thyroid cancer worldwide, according to GLOBOCAN 2012 (Figure 1), and the incidence of thyroid cancer has increased rapidly since 1999 [1,2]. Although the incidence of thyroid cancer has been increasing worldwide, the unprecedented rapid increase of thyroid cancer in Korea is rare in any country. The extremely high incidence of thyroid cancer in Korea is much higher than that of New Caledonia, which was the highest ever previously reported [1,3]. Most previous studies have suggested that the rapid increase in the incidence of thyroid cancer was due to early detection and increased diagnosis; in support of this, the number of cases of micro-sized thyroid cancer has increased [4,5]. Furthermore, although the incidence of thyroid cancer has increased, thyroid cancer mortality has stabilized [6,7]. Therefore, the Korea Central Cancer Registry (KCCR) has collected information on baseline characteristics and factors associated with thyroid cancer through medical chart review. The current epidemiologic study investigated the causes and changes in the clinical characteristics of thyroid cancer and is called the National Epidemiologic Survey of Thyroid cancer (NEST) study.

## DATA RESOURCE

### The Korea National Cancer Incidence Database

The NEST data resource was based on the Korea National Cancer Incidence Database (KNCI DB). The KCCR has collected data on nationwide cancer incidence for patients diagnosed since 1999 in Korea and has published cancer statistics since 2004 [8]. In 2002, the KCCR set up the KNCI DB by integrating some regional cancer registries and site-specific data from academic societies with the cancer incidence database of the KCCR [8]. Currently, the KNCI DB includes almost all incident cases of cancer in Korea [1]. In 2014, the coverage of the statistics on cancer incidence in the KNCI DB was estimated to be about 97.8% of the total cancer incidence based on the Ajiki method [1,9]. The microscopic verification percentage of the cancer statistics was approximately 90.0%, and the proportion of death certificates issued was only 1.2% in Korea [10].

## MEASURES

### The National Epidemiologic Survey of Thyroid cancer study

#### Questionnaire development

**Figure 2.**
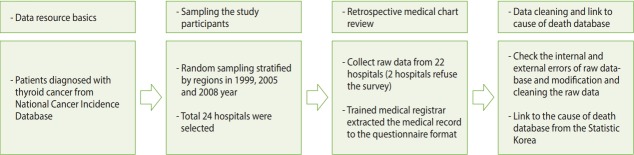
Summary of the National Epidemiologic Survey of Thyroid cancer study procedure.

The KCCR conducted the NEST in 2011 to investigate secular trends in the clinicopathologic features of Korean patients with thyroid cancer. To gather information on the diagnostic characteristics of patients with thyroid cancer, we conducted the NEST through retrospective medical chart review (Figure 2). Thyroid cancer incidence was defined as a newly-appearing topographical code of C73.9 according to the International Classification of Diseases, 10th edition. The questionnaire was developed based on a literature review and evaluated by experts from an academic society (The Korean Thyroid Association, The Korean Endocrine Society, The Korean Society of Pathologists, etc.). A pilot study was conducted to evaluate the feasibility and validity of the questionnaire. The content of the survey was finalized through the pilot study.

#### Sampling method

A 2-stage sampling method was used to obtain a nationally representative sample in the NEST study. To select representative patients with thyroid cancer, we obtained data on incident cases of thyroid cancer from the KNCI DB of the KCCR. We selected pools of thyroid cancer patients diagnosed in 1999, 2005, and 2008 from the KNCI DB. The 2005 data were sampled between 1999 and 2008 because thyroid cancer among women became the most frequently diagnosed malignancy in 2005. First, we randomly selected 24 hospitals using proportionate stratification, reflecting the number of patients stratified by regions in each year. Second, we randomly selected cases using proportionate stratification from the 24 hospitals selected in each year. We oversampled cases from 1999 and 2005 because there were insufficient thyroid cancer cases for analysis compared to the year 2008. The sampling proportion was 33% for 1999, 22% for 2005, and 11% for 2008 at the second stage, considering an approximate drop-out rate of 10%.

#### Selection of the study participants

**Figure 3.**
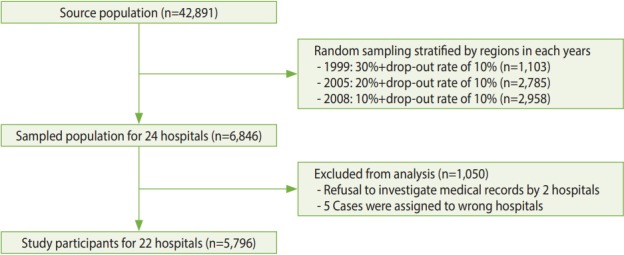
Flow chart for study participant selection.

Among the 42,891 participants who were diagnosed with thyroid cancer, 6,846 were included in the sampled population using the stratified random sampling method. Of these 6,846 participants, 1,045 cases (15.3%) were excluded because 2 hospitals refused to investigate their medical records, and 5 cases (0.1%) were excluded because of assignment error. Finally, 5,796 participants were included in the analysis (Figure 3). Informed consent was not obtained, but the presented data were anonymized, and the risk of identification was low.

#### Data collection

We collected data regarding age, gender, date of first diagnosis of cancer, date of death, date of the most valid examination for cancer [11], date of surgery, histologic type, the route of detection through which cancer was first detected (screening vs. clinical suspicion), multifocality, extrathyroidal invasion, method of cancer treatment, history of other comorbid diseases, and history of smoking and alcohol drinking. The date of first diagnosis of cancer indicates the date of the first consultation at, or admission to, a hospital for the cancer, the first diagnosis of the cancer by a physician, or the first pathology report and the most valid examination for cancer presents that the date on which the most valid test was performed to confirm the diagnosis of cancer. We also collected data on tumor, lymph node, metastasis (TNM) staging for thyroid cancer according to the sixth edition of the American Joint Committee on Cancer guideline published in 2002 [12].

The histological types of thyroid cancer were classified according to the International Classification of Diseases for Oncology, third edition (ICD-O-3) as papillary carcinoma (ICD-O-3 codes 8050, 8260, 8340–8344, 8350, and 8450–8460), medullary carcinoma (ICD-O-3 codes 8345 and 8510–8513), follicular carcinoma (ICD-O-3 codes 8290 and 8330–8335), anaplastic carcinoma (ICD-O-3 codes 8020–8035), and others (ICD-O-3 codes 8000–8005, 8337, 8346, and 8347) [13].

The NEST dataset was linked to the cause-of-death statistics from Statistics Korea (http://kostat.go.kr). Thyroid cancer patients were followed up through December 31, 2016. Five patients were lost to follow-up due to emigration or cancellation of resident registration. The cause-of-death data will be linked to the NEST dataset and updated every year. The research protocol for the present study was approved by the institutional review board of the National Cancer Center (no. NCC2017-0070).

## DATA RESOURCE USE

### Characteristics of study participants by year

Table 1 shows the baseline characteristics of the study participants according to the diagnosis of thyroid cancer each year. The mean age of thyroid cancer patients in this study was 46.9 years. The ratio of men to women was 1:5.5. According to the histologic type of cancer, the proportion of papillary thyroid carcinoma showed an increasing trend, while the other types of thyroid cancer showed decreasing trends (p for trend <0.01). The proportion of cases of distant metastasis showed a decreasing trend during the study period (p for trend <0.01). The mean tumor size also showed a decreasing trend during the study period (p for trend <0.01). The proportion of patients with lymph node involvement (p for trend <0.01) and distribution of TNM stages differed over time (p for trend <0.01). In particular, the proportion of patients with stage III thyroid cancer increased from 10.9% in 1999 to 22.2% in 2008, while the proportion of those with stage IV thyroid cancer decreased from 11.3% in 1999 to 5.8% in 2008 (p for trend <0.01).

### Number and causes of death among thyroid cancer patients

We followed thyroid cancer patients until December 31, 2016. During an average of 10.6 years of follow-up, 370 patients died (Table 2). Of these 370 deaths, 128 patients (34.6%) died from primary thyroid cancer, 127 patients (34.3%) died from other types of cancer, and 38 patients (10.3%) died from cardiovascular disease.

### Publications

Until now, there have only been 2 publications based on the NEST dataset [7,14]. The first is a study of changes in the prevalence of chronic lymphocytic thyroiditis among papillary thyroid cancer patients [14]. Chronic lymphocytic thyroiditis, or Hashimoto thyroiditis, is an autoimmune disease; notably, autoimmune diseases of the thyroid gland are a risk factor for thyroid cancer. Recently, some studies have reported that the incidence of Hashimoto thyroiditis has been increasing, along with thyroid cancer. In the NEST data, the age-standardized prevalence of chronic lymphocytic thyroiditis among thyroid cancer patients increased from 1999 to 2008. This finding suggests that in more recent years, the incidence of thyroid cancer was associated with factors related to autoimmune thyroid diseases or more detailed pathological examination. Another study estimated the difference in age-standardized incidence rates between 1999 and 2008 according to the route of tumor detection (screening detection vs. clinical detection) [7]. These results showed that the increase in the incidence of thyroid cancer in Korea was mainly due to over-detection resulting from the widespread utilization of sensitive imaging tools such as ultrasonography.

## STRENGTHS AND WEAKNESSES

The strength of the NEST cohort is that first, it represents the entirety of patients with thyroid cancer in Korea. Second, it also includes multiple variables, such as tumor size (mm), information on tumor stage, and method of treatment, that are not in the KCCR database. Various conversions are possible, because the information about tumor stage was collected using a collaborative stage data collection system (https://cancerstaging.org/cstage/). Third, it is possible to analyze patients’ prognosis and prognostic factors, because the NEST dataset has been linked to death data collected by Statistics Korea. The additional linkage allows researchers to conduct more specific analyses of relevant variables to obtain valuable results. This linked dataset has been used to analyze survival outcomes for thyroid cancer patients after diagnosis or according to certain variables, with some seeking to identify patient characteristics associated with specific outcomes.

There are some limitations to this study. First, our data may have been affected by misclassification bias regarding the route of detection. We reviewed medical records retrospectively. Although it was assumed that medical charts were unbiased records of patients’ information, there may be discrepancies between patients’ actual information and the medical information recorded on the chart. Indeed, Fouwels et al. [15] found that unhealthy lifestyle habits recorded in medical charts were underreported compared to the lifestyle habits that participants reported in their lifestyle questionnaire survey. Second, there were many missing variables, such as smoking history (missing, 12.5-45.2%), alcohol drinking (missing, 12.0-59.1%), or method of detection (missing, 13.1%). This missing information may result in bias. Third, some thyroid cancer cases were excluded due to refusal of the hospital to investigate or assignment error. However, we were able to re-calculate the number of non-responders for each stratum, because we knew the total number of incident cancer cases for each stratum. Therefore, we used post-stratification weights, and the effects of bias due to non-response units are expected to be minimal. Fourth, the NEST does not contain any information concerning the socioeconomic status, comorbidities, radiation exposure, or body mass index of participants, which are major risk or prognostic factors for thyroid cancer.

Despite these limitations, we expect the annual number of publications using the NEST dataset to increase. Publishable studies using the NEST dataset can cover a diverse range of topics. Potential researchers can examine the changes in characteristics associated with thyroid cancer by year, longitudinal patterns of treatment, and survival by prognostic factors. Regional comparisons have included evaluations of the impact of clinical evidence or guidelines, as well as health care policies.

## DATA ACCESSIBILITY

The KCCR developed the website for the NEST data (available from: http://kccrsurvey.cancer.go.kr/index.do). The NEST data are freely available. To download the NEST data, registration is required to access the website. After logging into the website, users click to request the NEST data, after which they have to sign a consent form, select the data, and write a brief research proposal. After approval by the staff, users can download the required data.

## Figures and Tables

**Table 1.** Baseline characteristics of study participants with thyroid cancer by year

Variables	Total	Year			p for trend^1^
		1999	2005	2008	
Total (n)	5,796	891	2,355	2,550	
Age (yr)	46.9±12.4	46.0±14.3	47.3±12.5	46.8±11.6	0.64
Tumor size (mm)	13.3±11.7	21.5±15.9	13.6±11.1	10.5±9.0	<0.01
Gender					0.13
Men	898 (15.5)	136 (15.3)	328 (13.9)	434 (17.0)	
Women	4,898 (84.5)	755 (84.7)	2,027 (86.1)	2,116 (83.0)	
Histologic type (carcinoma)					<0.01
Follicular	173 (3.0)	62 (7.0)	66 (2.8)	45 (1.8)	
Papillary	5,500 (94.9)	779 (87.4)	2,243 (95.2)	2,478 (97.2)	
Medullary	43 (0.7)	13 (1.4)	19 (0.8)	11 (0.4)	
Anaplastic	26 (0.5)	15 (1.7)	6 (0.3)	5 (0.2)	
Others	54 (0.9)	22 (2.5)	21 (0.9)	11 (0.4)	
Regional lymph node involvement					<0.01
No	2,466 (42.6)	268 (30.1)	1,012 (43.0)	1,186 (46.5)	
Yes	2,047 (35.3)	319 (35.8)	799 (33.9)	929 (36.4)	
Unknown	1,283 (22.1)	304 (34.1)	544 (23.1)	435 (17.1)	
Distant metastasis					<0.01
No	5,380 (92.8)	774 (86.9)	2,196 (93.3)	2,410 (94.5)	
Yes	34 (0.6)	15 (1.7)	14 (0.6)	5 (0.2)	
Unknown	382 (6.6)	102 (11.4)	145 (6.1)	135 (5.3)	
AJCC sixth edition stage					<0.01
I	3,038 (52.4)	428 (48.0)	1,249 (53.0)	1,361 (53.3)	
II	49 (0.9)	14 (1.6)	23 (1.0)	12 (0.5)	
III	1,036 (17.9)	97 (10.9)	373 (15.8)	566 (22.2)	
IV	426 (7.3)	101 (11.3)	178 (7.6)	147 (5.8)	
Unknown	1,247 (21.5)	251 (28.2)	532 (22.6)	464 (18.2)	

Values are presented as mean±standard deviation or number (%). AJCC, American Joint Committee on Cancer.

1 p for trends were tested by simple linear regression model for continuous variables and by the Mantel-Haenzel chi-square test for categorical variables.

**Table 2.** Number and causes of death among thyroid cancer patients

Characteristics	Thyroid cancer patients^1^
Total (n)	5,796
Men	898 (15.5)
Women	4,898 (84.5)
Follow-up period (mean±SD, yr)	10.6±3.3
Censored cases	5 (0.1)
No. of deaths (n=370)	370 (6.4)
Men	109 (29.5)
Women	261 (70.5)
Causes of death (ICD-10 code)	
Thyroid cancer (C73)	128 (34.6)
Other types of cancer (C00 – C97, except C73)	127 (34.3)
Cardiovascular disease (I00 – I99)	38 (10.3)
Other causes of death	77 (20.8)

Values are presented as number (%). SD, standard deviation; ICD-10, International Classification of Diseases, 10th edition.

1 Thyroid cancer patients were followed up through December 31, 2016.
